# Effects of bifidobacterium lactis bb12 probiotic consumption on streptococcus mutans levels: a systematic review of randomized clinical trials

**DOI:** 10.21142/2523-2754-1402-2026-287

**Published:** 2026-04-04

**Authors:** Emily Gabriela Acuña Ponce de León, Brenda María Zapata Cedrón, Carol Magaly Cárdenas Flores

**Affiliations:** 1 School of Dentistry, Universidad Cientifica del Sur. Lima, Peru. 100049134@cientifica.edu.pe 100046646@cientifica.edu.pe Universidad Científica del Sur School of Dentistry Universidad Cientifica del Sur Lima Peru 100049134@cientifica.edu.pe 100046646@cientifica.edu.pe; 2 Department of Paediatric Dentistric, School of Dentistry, Universidad Cientifica del Sur. Lima, Peru. ccardenasf@cientifica.edu.pe Universidad Científica del Sur Department of Paediatric Dentistric School of Dentistry Universidad Cientifica del Sur Lima Peru ccardenasf@cientifica.edu.pe

**Keywords:** Bifidobacterium, Bifidobacterium animalis, Bifidobacterium lactis bb12, Probiotics, Streptococcus mutans, dental caries, Bifidobacterium, Bifidobacterium animalis, Bifidobacterium lactis BB12, probióticos, Streptococcus mutans, caries dental

## Abstract

**Introduction:**

One of the main bacteria related to dental caries is Streptococcus mutans, and currently, various studies are evaluating how to decrease its count. The effect and efficacy of probiotic consumption as a method to inhibit this bacterium are currently being analyzed.

**Objective:**

To evaluate the effect of the probiotic *Bifidobacterium lactis* BB12 (BB12) in its different presentations on *Streptococcus mutans* (SM) levels in children and adults.

**Materials and Methods:**

An electronic search was conducted in the PubMed, Scopus, EBSCO, and EMBASE databases to find randomized clinical trials that evaluated the effect of the probiotic BB12 on SM levels.

**Results:**

Studies demonstrated an association between the consumption of BB12 and the reduction of SM count.

**Conclusions:**

Further studies are needed to analyze the effect of the probiotic BB12 on SM, particularly studies investigating larger study populations and conducted over longer study periods.

## RESUMEN

**Introducción:**

Una de las principales bacterias relacionadas con la caries dental es *Streptococcus mutans*, y actualmente se están realizando diversos estudios para evaluar cómo disminuir su recuento. Se están analizando el efecto y la eficacia del consumo de probióticos como método para inhibir esta bacteria.

**Objetivo:**

Evaluar el efecto del probiótico *Bifidobacterium lactis* BB12 (BB12) en sus diferentes presentaciones sobre los niveles de *Streptococcus mutans* (SM) en niños y adultos.

**Materiales y métodos:**

Se realizó una búsqueda electrónica en las bases de datos PubMed, Scopus, EBSCO y Embase para encontrar ensayos clínicos aleatorizados que evaluaran el efecto del probiótico BB12 sobre los niveles de SM.

**Resultados:**

Los estudios demostraron una asociación entre el consumo de BB12 y la reducción del recuento de SM.

**Conclusiones:**

Se necesitan más estudios para analizar el efecto del probiótico BB12 sobre SM, en particular estudios con poblaciones más amplias y realizados durante períodos de estudio más prolongados.

## INTRODUCTION

The World Health Organization (WHO) states that 2.3 billion people worldwide suffer from dental caries ^1^. This chronic, multifactorial, and progressive disease results from a dysbiosis in the oral cavity. When this imbalance occurs, acid-producing bacteria begin to degrade the dental surface due to demineralization, thus creating “cavities” ^2^. Dysbiosis is defined as an imbalance in the microbial colonies that have colonized a mucosal surface, such as the oral cavity ^3^. The combined function of microbiota can lead to either a state of homeostasis or, conversely, a disequilibrium, which may manifest as signs of health or disease ^4^. Epidemiological studies indicate that 35% of the adult global population suffers from untreated carious lesions^5^. Additionally, the WHO notes that 520 million children under the age of 12 suffer from caries ^1^. One study estimates that approximately 76 % of Peruvian children under the age of 5 and 85% under the age of 11 have caries ^6^.

Currently, it continues to be considered a significant problem. The Institute for Health Metrics and Evaluation emphasizes this disease, noting that in 32 years, dental caries has only decreased by 4% globally, despite all advances and technologies in this área ^7^.

At the present, dentistry aims to treat these carious lesions, intervene in their etiology, and prevent them, starting with the main contributor ^8^. Over time, it has been described that the main bacterium responsible for producing this disease is Streptococcus mutans. This bacterium is also related to potential infectious endocarditis ^9^ and indirectly related to other extraoral pathologies, such as cardiovascular disorders and strokes ^10^. In addition to acidifying the oral environment, this bacterium strongly adheres to the dental substrate, thus promoting the formation of a biofilm that is difficult to eliminate mechanically. Consequently, efforts are made to prevent this colonization through the appropriate dosing of selective, non-digestible, non-pathogenic live microorganisms called probiotics. These probiotics will strongly adhere to the oral cavity, displacing cariogenic and periodontopathogenic bacteria by taking up their adhesion sites and producing bacteriocins that will cause their death or inhibit their proliferation ^7^^-^^9^.

Diet is one of the factors triggering dental caries, which has led to interest in various foods, such as probiotics ^11^^-^^12^. According to the National Center for Complementary and Integrative Health, probiotics are defined as live microorganisms that are beneficial to health when consumed or applied to the body ^13^. They can be found in yogurt and other fermented foods and/or dairy products, such as pickles, ice cream, dietary supplements, and beauty products ^13^. It is established that these microorganisms have anti-inflammatory properties and could also provide benefits to the immune system without reported side effects ^14^. Currently, a precise mechanism by which they act is not known; however, it is suggested that they adhere to epithelial surfaces, produce immunostimulation, and also have antagonistic activity against pathogenic microorganisms ^15^. Some of the most common ones include lactobacilli, bifidobacteria, saccharomyces, streptococci, enterococci, etc. ^16^.

There is currently scientific evidence about probiotics and their multiple benefits to oral and systemic health ^2^^,^^17^^-^^24^. Furthermore, a systematic review published in 2014 showed that probiotics could influence the reduction of SM, preventing the development of carious lesions ^2^. Various studies are currently investigating the effect and efficacy of the probiotic BB12 in children and adults ^25^^-^^32^, to observe and analyze whether this method, administered in adequate amounts, is effective in the proliferation of SM without any adverse effects ^31^. However, to our knowledge, there is not a broad, updated literature focusing on the inhibition of SM with BB12 as the probiotic of choice. Therefore, the main objective of this systematic review is to evaluate the effect of the probiotic BB12 in inhibiting the proliferation of SM. The objective was to evaluate the effect of the BB12 probiotic on inhibiting the proliferation of SM.

## MATERIAL AND METHODS

### Protocol and Registration

A systematic review based on PRISMA guidelines and registered in PROSPERO with registration code: CRD42023404164.

### Search Strategy

Three reviewers (GA), (BZ), and (CC) conducted a systematic search in the following databases (EMBASE, Medline/PubMed, Scopus, EBSCO) to find randomized clinical trials on *Bifidobacterium lactis* BB12 probiotic and its inhibitory effect on *Streptococcus mutans*.

The search strategy used controlled vocabulary terms MeSH combined with Boolean operators (AND, OR) based on PICOS elements (Table 1), developed initially in PubMed and subsequently adapted to other databases. Additionally, a manual search was conducted in high-impact journals such as Medline, Scopus, Science Direct, Embase, etc. Keywords used included *Bifidobacterium*, *Bifidobacterium animalis*, *Bifidobacterium lactis*, *Bifidobacterium lactis BB12*, Probiotics, Synbiotics, Dietary Supplements, *Streptococcus mutans*.

**Table 1:** PICOS elements

Acronym	Definition	Description
P	Population	Children and adults between 6 and 40 years old
I	Intervention	Oral administration of the probiotic *Bifidobacterium lactis* BB12 in any form (yogurt, milk, tablets, ice cream)
C	Comparison	Placebo
O	Outcome	Levels of *Streptococcus mutans*, methods of bacterial quantification
S	Study Design	Randomized clinical trials

Search Consolidation: ("Bifidobacterium"[MeSH Terms] OR "Bifidobacterium"[Title/Abstract] OR "bifidobacterium animalis"[MeSH Terms] OR "bifidobacterium animalis"[Title/Abstract] OR "bifidobacterium lactis"[Title/Abstract] OR "bifidobacterium lactis bb12"[Title/Abstract]) AND ("Probiotics"[MeSH Terms] OR "Probiotics"[Title/Abstract] OR "Synbiotics"[MeSH Terms] OR "Synbiotics"[Title/Abstract] OR "dietary supplements"[MeSH Terms] OR "dietary supplements"[Title/Abstract]) AND ("streptococcus mutans"[MeSH Terms] OR "streptococcus mutans"[Title/Abstract]) (Table 2)

**Table 2:** Search strategy

MedLine/PUBMED (24 /04 /23)
n = 36
("Bifidobacterium"[MeSH Terms] OR "Bifidobacterium"[Title/Abstract] OR "bifidobacterium animalis"[MeSH Terms] OR "bifidobacterium animalis"[Title/Abstract] OR "bifidobacterium animalis"[MeSH Terms] OR "bifidobacterium lactis"[Title/Abstract] OR "bifidobacterium lactis bb12"[Title/Abstract]) AND ("Probiotics"[MeSH Terms] OR "Probiotics"[Title/Abstract] OR "Synbiotics"[MeSH Terms] OR "Synbiotics"[Title/Abstract] OR "dietary supplements"[MeSH Terms] OR "dietary supplements"[Title/Abstract]) AND ("streptococcus mutans"[MeSH Terms] OR "streptococcus mutans"[Title/Abstract])
Scopus (24/04/23)
n = 3
("Bifidobacterium" OR "bifidobacterium animalis" OR "bifidobacterium lactis" OR "bifidobacterium lactis bb12")AND ("Probiotics" OR "Synbiotics” OR "dietary supplements") AND ( "streptococcus mutans")
Embase (25 /04 /23)
n = 9
("Bifidobacterium" OR "bifidobacterium animalis" OR "bifidobacterium lactis" OR "bifidobacterium lactis bb12")AND ("Probiotics" OR "Synbiotics” OR "dietary supplements") AND ( "streptococcus mutans")
EBSCO (27/04 /23)
n = 11
("Bifidobacterium" OR "bifidobacterium animalis" OR "bifidobacterium lactis" OR "bifidobacterium lactis bb12")AND ("Probiotics" OR "Synbiotics” OR "dietary supplements") AND ( "streptococcus mutans")
Google Schoolar (27 /04 /23)
n = 2
("Bifidobacterium" OR "bifidobacterium animalis" OR "bifidobacterium lactis" OR "bifidobacterium lactis bb12")AND ("Probiotics" OR "Synbiotics” OR "dietary supplements") AND ( "streptococcus mutans")

A manual search was performed by examining references used in the studies included in this systematic review. Additionally, searches were conducted in the clinical trial registry database: clinicaltrials.gov; Cochrane Central Register of Controlled Trials, as well as grey literature such as Google Scholar and high-impact journals like "Caries Research", "Clinical Oral Investigations", "European Journal of Orthodontics", "Oral health & preventive dentistry", "The Journal of Clinical Pediatric Dentistry", "European archives of paediatric dentistry", and "Journal of Applied Oral Science".

### Inclusion Criteria

Randomized clinical trials (RCTs) without age, gender, or ethnicity restrictions of participants, evaluating the inhibitory effect of BB12 probiotic on SM, published between January 2005 and March 2023. 

### Exclusion Criteria

Studies involving patients with diseases that could interfere with probiotic function and hence their count, such as gastritis, immunosuppressed individuals, smokers, etc.

Similarly, studies involving patients with fixed orthodontic appliances, which could alter oral environment readings, were excluded. Studies analyzing probiotic use with any enhancer that could potentiate its effect and not evaluating its capability on its own were excluded. Studies with insufficient information on study design.

### Data Collection

61 articles were obtained through bibliographic search by (GA, BZ, and CC), exported to Mendeley 2.76.0 (Elsevier, New York, USA), where 12 duplicate studies were removed. Two researchers (GA) and (BZ) evaluated titles and abstracts according to established inclusion criteria, selecting 12 articles. Subsequently, the full text of selected studies was independently reviewed, and 7 articles were chosen for inclusion in this review. Disagreements were resolved through consensus with a third reviewer (CC). This entire process was conducted using the Rayyan program.

### Selection Diagram of Articles Included in this Study

**Figure 1:**
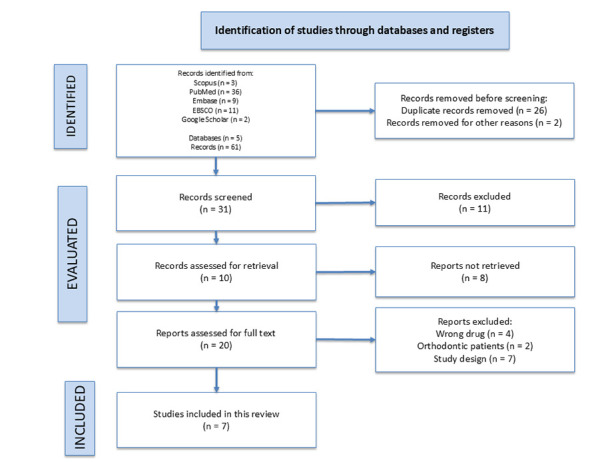
Selection diagram of the articles included in this study

With the algorithm, a total of 61 articles were identified, of which 26 were excluded: 2 for being duplicates, 10 for not meeting the PICOS question, and 10 because the full article version could not be found; these versions were requested, where only 2 of these articles could be obtained. Out of the total articles considered for full-text review, 20 articles were selected, and 13 articles were excluded. Seven of these were discarded for not meeting the required study design, 4 for analyzing the wrong drug, and 2 for evaluating patients with orthodontics. Finally, 7 RCT studies were included for qualitative analysis of the information. (Figure 1)

### Standardization Criteria Process

Using Rayyan, the Cohen's Kappa coefficient test was conducted to measure agreement between reviewers GA and BZ, resulting in values ranging from 0.00 (poor agreement strength) to 1.00 (almost perfect agreement).

### Data Extraction

GA and BZ began data extraction for this study, adapting the Cochrane Consumers and Communication Review Group's model using a predefined xlsx spreadsheet (Microsoft Excel). Two reviewers (GA, BZ) independently entered the following data from included studies: Authors, year, country, study type, participant age and number, trial and control interventions, SM counts in saliva, SM counts in dental plaque, presentation of probiotic or product (yogurt, pill, etc.), daily dose, total dose (daily dose in mg multiplied by probiotic consumption time in colony forming units [CFU]), frequency and duration of consumption, main results, adverse events, and principal conclusions.

A third investigator (CC) was involved in the case of disagreements during selection to resolve discrepancies. Unclear or undescribed data were requested via email to authors for clarification.

### Data Analysis and Evaluation

 Synthesized description of extracted data was consistently and orderly described to facilitate development of evaluation tables. Heterogeneous results were obtained, precluding meta-analysis. 

### Risk of Bias

Reviewers GA and BZ assessed risk of bias in included studies using Rob 2.0 tool, through five domains including "signaling questions" to detect methodological gaps. Data resulted in a bias judgment with responses categorized as low, high, or some concerns. Disagreements required involvement of a third investigator (CC) ^21^.

## RESULTS

### General Characteristics of Included Studies

Most of the articles correspond to studies published in 2015, with variations such as the study by Esber et al., which was published in 2008 ^(25, 26)^. Additionally, the majority were conducted in Asia and the Middle East. To a greater extent, they were double-blind trials whose general objective was to analyze the effect of probiotic consumption on SM. All studies in relation to the experimental group consumed BB12 probiotic in some proportion, usually in presentations such as yogurt or ice cream, while the control group consumed these types of products without any proportion of probiotics. Most studies provided similar and heterogeneous sample sizes, except for the study by Salama ^31^. However, these varied in terms of age category. The follow-up time was similar for almost all studies, most recording only one analysis period, except for the study by Ali Nozari et al. ^29^ which had 4 follow-up periods, and the study by Singh et al., which had a 7-day follow-up ^27^ (Table 3).

**Table 3:** General characteristics of the selected articles (n = 7)

Author	Year	Country	Study type	Objective	experimental Group	control Group	Participant Ag	CFU Count		N. ofparticipants	Follow-up time
								Base	Post		
Caglar *et al*. ^25^.	2005	Turkey	RCT crossover	To examine whether short-term consumption of yogurt wiht bifidobacterium would affect salivary levels of SM and *Lactobacillus* in Young adults	Yogurt with probiotic ^(^200g)	Yogurt without probiótico	21-24 years	-	-	21	9 weeks
Caglar *et al*. ^26^.	2008	Turkey	RCT crossover	Evaluate the effects of probiotics in yogurts on SM and Lactobacillus	Ice cream with probiotic	Ice cream without probiotic	20 years	-	-	24	41 days
Sigh *et a*l. ^27^.	2011	Poland	RCT	Comparing the levels of SM and Lactobacillus in the saliva of schoolchildren, before and after consumption of probiotics and control ice cream	Vanilla Ice cream with probiotic (54 g)	Ice cream without probiotic	12-14 years	-	-	40	41days
Nagarajappa *et al*. ^28^.	2015	India	RCT Double-blind	Examining the effect of chocolate ice cream containing *Bifidobacterium lactis* on salivary *Streptococcus mutans* salivales.	Chocolate ice cream with 42g of the probiotic	Chocolate Ice cream without probiotic	18-22 years	8,1648 x 10 6 CFU/mL	2,4 x 10 6 CFU/mL	30	18 days
Nozari *et al*. ^29^.	2015	Iran	CRT Crossover	To evaluate the effect of probiotic yogurt consumption on salivary cariogenic microflora in children	200g of probiotic yogurt	Yogurt conventional	6-12 years	6,0083 × 104	5,0416 × 104	49	8 weeks
Zare *et al*. ^31^.	2015	Iran	RCT Double-blind	To examine the effetc of chocolate ice cream containing *Bifidobacterium lactis* on salivary *Streptococcus mutans*.	Probiotic yogurt with 10(6) CFU/mL	Conventional yogurt	18-30 years	4,08 +- 3,92 CFU	3,28 +- 3,46 CFU	60	2 weeks
Salama *et al*. ^30^.	2018	Egypt	RCT Double-blind	Evaluating the effects of probiotics in yogurts on *Streptococcus mutans* and *Lactobacillus*	Yogurt with probiotic	Yogurt without probiotic	3-6 years	211,89 ± 18,632 CFU/ml	77,94 ± 4,862 CFU/mL	350	14 days

RCT = Randomized Clinical Trial; CFU = Colony Forming Units; CFU/mL = Colony Forming Units per milliliter

### Effects of Bifidobacterium lactis BB12 Probiotic on Streptococcus mutans 

It can be noted that only the studies by Ahmad Zare, Salama, and Mahmoud evaluated the DMFT index before procedures were performed, while none of the included studies analyzed SM levels at the level of dental plaque. All studies showed statistically significant reductions for the experimental group in relation to SM level counts compared to the control group, where no study showed significance in the control group. (Table 4)

**Table 4:** Effects of the probiotic Bifidobacterium lactis BB12 on Streptococcus mutans in randomized crossover clinical trials

Autor, año	Experimental Group			Control Group					Bacterial Plaque Levels
	DMFT INDEX	Mean SD of SM		P Value	Bacterial Plaque Levels	DMFT Index	Mean SD of SM			
		Base	Post- Int				Base	Post-Int		
Nagarajappa, 2015	-	8,1648x10(6) ±1,5240x10(7)	2,19x10(5) ± 7,1572x10(5)	0,001*	-	-	3,4277x10(6) ± 6,6035 x 10(6)	5,2119x10(6) ± 1,0006x10(6)	-
Ahmad Zare, 2015	2,2±0,7	4,08±3,92	3,28±3,26	0,001*	-	2,2 ± 0.9	4,13 ± 3,97	3,64 ± 3,43	-
Salama y Mahmoud, 2018	5,507 ± 1,014	211,89 ±18,632	77,94±4,862	0,01*	-	4,558 ± 0,977	215,12 ± 20,214	217,37 ± 19,997	-

DMFT index = Index of missing and filled carious teeth; SD = Standard deviation; SM = *Streptococcus mutans*; P value < 0.05 = Statistically significant value.

### Effects of Bifidobacterium lactis BB12 Probiotic on Streptococcus mutans in Cross-Over Randomized Clinical Trials

Almost all studies showed results categorized according to key UFC/ml scores (0, 1, 2, 3), except for the study by Ali Nozari, which evaluated results based on periods. All studies in the table reported a statistically significant reduction in SM levels at the end of the intervention, with most resulting in an increase in scores 0 and 1, accompanied by a decrease in scores 2 and 3. The study by Esber et al provided noteworthy results, where scores 2 and 3 became null. (Table 5).

**Table 5:** Effects of the probiotic *Bifidobacterium lactis* BB12 on *Streptococcus mutans*

Autor	Control (n)								Probiotic(n)								P value*	
	Base				Post-Int				Base				Post-Int							
	0	1	2	3	0	1	2	3	0	1	2	3	0	1	2	3	
Caglar, 2008	4	8	8	4	9	9	6	0	0	10	10	3	8	15	0	0	0.05*
Singh, 2011	3	6	15	15	4	5	17	13	2	6	15	16	4	9	16	10	0.003*
Caglar, 2005	1	6	11	3	2	9	7	3	2	6	10	3	6	4	10	1	0.05*
Control										Probiotic									
	S1		S2		S3		S4		S1		S2		S3		S4			
Nozari, 2015	129200		120160		112080		64960		60083		49750		55416		50416		0.003	

Score 0: < 10(3), Score 1: <10(4), Score 2: 10 (4) - 10 (5), Score 3: >10 (5)

### Risk of Bias Analysis in Randomized Clinical Trials

**Figure 2:**
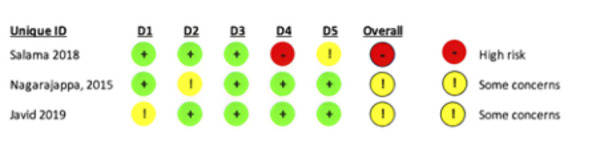
Risk of bias assessment of non-crossover clinical trials included in the study.

From the analysis conducted, one study presented a high risk of bias, and two studies raised some concerns. Regarding the randomization method, the studies by Salama 2018 and Nagarajappa 2015 mentioned having performed this process; however, they did not specify the method used, while the study by Javid raised some concerns in this aspect. The study by Salama 2018 showed some inconsistencies in terms of outcome measures due to insufficient information (Fig. 2).

### Risk of Bias Analysis in Cross-Over Randomized Clinical Trials

**Figure 3:**
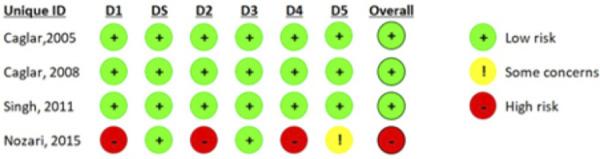
Risk of bias analysis of randomized crossover clinical trials

The study by Nozari et al. shows a high risk of bias due to lack of information in the randomization process and blinding of examiners; however, the article by Singh et al. appropriately describes all its randomization methodology, resulting in a low-risk outcome, as did the article by Caglar et al. Although there was a lack of information in the randomization process of individuals, this was compensated by the allocation concealment described (Fig. 3).

## DISCUSSION

This systematic review investigated the inhibitory effects of the probiotic BB12 on the bacterium SM, in its various dairy presentations, as an adjunct in dental caries prevention. The oral cavity harbors a symbiotic balance of microorganisms; however, an accumulation of cariogenic bacteria, such as SM, leads to dysbiosis. This dysbiosis is influenced by several additional factors including the host, time, substrate, and bacteria themselves.

Initially, we can analyze that most authors (Nagarajappa et al., Zare et al., Salama et al., Caglar et al., Singh et al.) report a significant decrease in SM counts (p<0.001, p<0.001, p<0.001, p<0.009, p<0.003 respectively) after consuming a product containing probiotic BB12 compared to consuming a placebo ^25^^,^^26^^,^^28^^,^^30^^,^^31^. However, studies such as that of Nozari et al., which did not report a significant decrease in SM counts in the short term (p>0.383) ^29^. Despite this, differences in results could be attributed to the small sample size, age range variations, and the study methodologies presented. Caglar et al. 2008; showed robust results over a 2-week period in categories 3 and 4, finding nearly total inhibition of SM (P<0.05) ^25^^,^^26^.

Half of the studies analyzed the count in UFC/mL, such as Nagarajappa et al., Zare et al., Salama et al. ^28^^,^^30^^,^^31^ while others like Nozari et al., Caglar et al., and Singh et al., categorized counts using standardized proportions based on ascending scores from 1 to 4, providing insight into the reduction of SM ^25^^,^^26^^,^^29^^,^^27^. It's worth noting that these studies were crossover randomized controlled trials (RCTs), unlike those of Nagarajappa et al. and Zare et al., which were non-crossover RCTs. For analytical purposes, this study evaluated both designs separately ^28^^,^^31^.

This research demonstrates how the administration vehicle of the selected RCTs for the probiotic was not standardized but instead differed in commercial presentation (yogurt and ice cream). However, it was not possible to compare the results of both vehicles due to the nature of the analyzed RCTs. It was also observed that the commercial presentation was easy to ingest, as evidenced by the study of Nagarajappa, which reported no difficulty in providing the product and that participants could not differentiate between the experimental (probiotic ice cream) and the placebo. 28 An important finding reported by Caglar et al. was a significant reduction in SM counts using small proportions of the BB12 probiotic ^25^^,^^26^.

Laleman et al. suggests that the effects of probiotic bacteria emerge after continuous consumption. Therefore, it can be inferred that ceasing consumption of this product will nullify its effects. This underscores the importance of conducting studies with longer follow-up periods ^2^. A limitation of the analyzed studies may be that most analyzed results over short periods, typically 1 to 2 weeks.

Despite the findings related to oral health, it has been observed that the use of this probiotic not only benefits oral health, improving conditions such as periodontitis, gingivitis, and halitosis, but also addresses systemic health issues. For instance, Rezazadeh et al. found a decrease in LDL-C levels in diabetic patients following consumption of the BB12 probiotic ^13^.

Nagarajappa et al. study exhibited lower risk of bias, attributed to their detailed description of randomization procedures, allocation concealment, and participant/result blinding, like Singh et al.'s approach ^27^^,^^28^. In contrast, Nozari et al. merely reported performing these procedures without specifying tools used or addressing sample size discrepancies, leading to a high risk of bias ^29^. Concerns also arose in studies by Caglar et al., Salama et al., and Zare et al. due to insufficient data on participant blinding ^25^^,^^26^^,^^30^^,^^31^.

## CONCLUSIONS

The consumption of dairy products containing the probiotic BB12 was effective in reducing levels of SM. It was observed that the average duration of probiotic consumption was 4 weeks. However, further studies are needed to analyze the probiotic's effect on SM, particularly studies involving larger study populations and longer study durations.
